# Genetic mutations of *GJB2* and mitochondrial *12S rRNA* in nonsyndromic hearing loss in Jiangsu Province of China

**DOI:** 10.1186/1479-5876-11-163

**Published:** 2013-07-04

**Authors:** Qinjun Wei, Shuai Wang, Jun Yao, Yajie Lu, Zhibin Chen, Guangqian Xing, Xin Cao

**Affiliations:** 1 Department of Biotechnology, School of Basic Medical Science, Nanjing Medical University, Hanzhong Road No.140, Nanjing 210029, P.R. China; 2 Department of Otorhinolaryngology, The First Affiliated Hospital of Nanjing Medical University, Guangzhou Road No.300, Nanjing 210029, P.R. China

**Keywords:** Nonsyndromic hearing loss, *GJB2*, Mitochondrial *12S rRNA*, Gene mutation

## Abstract

**Background:**

Hearing loss is caused by several environmental and genetic factors and the proportion attributed to inherited causes is assumed at 50 ~ 60% . Mutations in *GJB2* and mitochondrial DNA (mtDNA) *12S rRNA* are the most common molecular etiology for nonsyndromic sensorineural hearing loss (NSHL). The mutation spectra of these genes vary among different ethnic groups.

**Methods:**

To add the molecular etiologic information of hearing loss in the Chinese population, a total of 658 unrelated patients with NSHL from Jiangsu Province of China were selected for mutational screening including *GJB2* and mtDNA *12S rRNA* genes using PCR and DNA sequencing technology. As for controls, 462 normal-hearing individuals were collected.

**Results:**

A total of 9 pathogenic mutations in the *GJB2* and 7 pathogenic mutations in the *12S rRNA* gene were identified. Of all patients, 70 had monoallelic *GJB2* coding region mutation in the heterozygous state, 94 carried two confirmed pathogenic mutations including 79 homozygotes and 15 compound heterozygotes. The 235delC appears to be the most common deafness-causing *GJB2* mutation (102/658, 15.50% ). No mutations or variants in the *GJB2* exon1 and basal promoter region were found. In these patients, 4 subjects carried the m.1494C > T mutation (0.61% ) and 39 subjects harbored the m.1555A > G mutation (5.93% ) in mtDNA *12S rRNA* gene. A novel sequence variant at m.1222A > G in the *12S rRNA* gene was identified, which could alter the secondary structure of the 12S rRNA.

**Conclusion:**

The mutation spectrum and prevalence of *GJB2* and mtDNA *12S rRNA* genes in Jiangsu population are similar to other areas of China. There are in total 31.46% of the patients with NSHL carry deafness-causing mutation in *GJB2* or mtDNA *12S rRNA* genes. Mutation in *GJB2* gene is the most common factor, mtDNA *12S rRNA* also plays an important part in the pathogenesis of hearing loss in Jiangsu Province areas. The m.1222A > G was found to be a new candidate mutation associated with hearing loss. Our results indicated the necessity of genetic screening for mutations of these genes in Jiangsu patients with NSHL.

## Introduction

Hearing loss is caused by several environmental and genetic factors, and the proportion attributed to inherited causes is assumed at 50-60% [1]. About 70% of which are nonsyndromic hearing loss (NSHL) since hearing loss is the only symptom. Genetic hearing loss of nonsyndromic form can follow a pattern of autosomal dominant (DFNA), autosomal recessive (DFNB), or X-linked recessive, and mitochondrial inheritance [2].

To date, there are 134 genetic loci and 80 different genes that have been described for NSHL in human [3]. It is believed that alterations in several members of the connexin protein family and mtDNA *12S rRNA* contribute to the development of the majority of genetic hearing losses, in which connexin 26 (*GJB2*) gene mutations are particularly an important cause of NSHL and mainly linked to pattern of autosomal recessive, and the single-nucleotide alteration mtDNA m.1555A > G has been identified as a major cause of aminoglycoside-induced NSHL [4-8].

It is estimated that there are approximately 20 million babies born every year in China, of whom about 30,000 are expected to have congenital hearing loss [9]. Carrier frequencies of some mutational hot spots associated with NSHL from different areas and ethnic backgrounds have been reported [10-15], but the molecular etiology of NSHL in the Chinese Jiangsu Province population has not been investigated systematically. To further extend the epidemiological data of common gene mutations in Chinese population, we screened the *GJB2* and mitochondrial *12S rRNA* genes to determine the etiology of hearing loss in Jiangsu Province.

## Materials and methods

### Subjects and audiological examinations

A total of 658 unrelated hearing loss subjects from 6 typical cities of Jiangsu Province, China were collected. All the individuals were recruited to participate in this study by signing a written informed consent as approved by the Ethical Committee of Nanjing Medical University for Human Studies, and to donate a peripheral blood sample. The protocol was reviewed and genetic studies were conducted on two groups: a case group with moderate to profound and NSHL (n = 658) aged 2–45 (11.3 ± 2.6) years, and a control group (n = 462) with normal hearing aged 8–34 (11.5 ± 2.9) years. The female–male ratio of these groups are 344/314 and 235/227, respectively. The blood samples of 658 cases were obtained from a panel of sporadic hearing loss individuals collected in the Otological Clinics between January 2008 and July 2012, and the 462 control blood samples were gotten from a panel of unaffected individuals in Jiangsu province. All subjects were Han Chinese in origin, and were evaluated through otological examination and audiological evaluations including pure-tone audiometry (Madsen Orbiter 922), immittance (Madsen Zodiac 901), auditory brainstem response (ABR) (Interacoustic EP25), and transient evoked otoacoustic emissions (Madsen Celesta 503). Hearing loss is defined by the level of hearing loss in the better ear for pure-tone threshold average in the speech frequencies 0.5, 1, 2, and 4 k HZ. Hearing loss of 26-40 dB was considered mild; 41-60 dB, moderate; 61-80 dB, severe, and more than 80 dB, profound.

### Mutational analysis of GJB2 and mitochondrial 12S rRNA genes

The mutations on the *GJB2* and mtDNA *12S rRNA* genes were selected for molecular analysis in all 658 NSHL cases and 462 normal controls. The genomic DNA was isolated from peripheral blood leukocytes of all participants using Puregene DNA Isolation Kits (Watson Biotechnologies Inc, Shanghai China). The quality and quantity of purified genomic DNA were determined by gel electrophoresis (0.8% agarose) and spectrophotometry.

The coding exon (exon 2) and flanking intronic regions of *GJB2* gene were amplified by PCR using primers F (5′TTGGTGTTTGCTCAGGAAGA3′) and R (5′ GGCCTACAGGGGTTT CAAAT 3′). The *GJB2* exon 1, its flanking donor splice site and the *GJB2* basal promoter were amplified with the primers F (5′ TGGGGGCACTTGGGGAACTCA 3′) and R (5′ GCAGAAACGCCCGCTCCAGAA 3′). DNA fragments of subjects spanning the mtDNA *12S rRNA* gene were also amplified by PCR with primers: P1 F (5′ CTCCTCAAAGCAATACACTG 3′) and R (5′ TGCTAAATCCACCTTCGACC 3′); P2 F (5′ CGATCAACCTCACCACCTCT 3′) and R (5′ TGGACAACCAGCTATCACCA 3′).

All the PCR products were purified on QIA-quick spin columns (Qiagen, Valencia, CA) and subjected to direct sequencing by BigDye Terminator Cycle Sequencing kit (version v.3.1) and ABI genetic analyzer 3730 (Applied Biosystems, Foster City, CA, USA) attached Sequencing Analysis software (version 3.7). The resultant sequence data of *GJB2* gene were compared with the reference sequence of wild type *GJB2* (GenBank No. NG_008358.1) and the genotypes of *GJB2* gene in all subjects were subsequently analyzed. PolyPhen-2 (http://genetics.bwh.harvard.edu/pph2/) and SIFT (http://sift.jcvi.org/) were used to predict the effect of previously reported and novel missense changes on protein function. The sequencing data of mtDNA *12S rRNA* gene were compared with the updated consensus Cambridge reference sequence (GenBank No. AC_000021.2) to identify mtDNA variants. The uniqueness of each mutation was evaluated by comparison with the MITOMAP, and the Uppsala mtDB database.

### Phylogenetic analysis and structural analysis of mitochondrial 12S rRNA

A total of 22 primates’ mtDNA *12S rRNA* sequences were selected to analyze the interspecific difference by ClustalW. The additional file lists the mammalian species and the accession numbers of the mtDNA *12S rRNA* (Additional file 1: Table S1). The conservation index (CI) was calculated by comparing the human nucleotide variants with other 21 primates. The CI was then defined as the amount of species from the list of 22 different primates that have the wild-type nucleotide at that position.

Pathogenicity of the novel variants was also evaluated by predicting the secondary structures of human mtDNA *12S rRNA* transcripts with or without the variant using the RNAdraw program (RNAdraw V1.1, http://www.rnadraw.com).

## Results

### GJB2 gene mutations

As shown in Table 1, there were totally fifteen kinds of different *GJB2* variations detected in the patients and controls. Among these, one variant c.257C > G (T86R) in coding region was novel pathogenic mutation that was identified in two deaf siblings compound with c.605ins46 mutation in the exon 2. The two siblings came from a Chinese Han family and suffered from prelingual and nonsyndromic hearing loss. The c.257C > G (T86R) mutation was also predicted to be damaging by PolyPhen-2 and SIFT. Eight variants including c.35delG, c.109G > A, c.176del16, c.235delC, c.299delAT, c.504insGCAA, c.605ins46 and c.608TC > AA were pathological mutations that has been reported previously. Four nucleotide changes containing c.79G > A, c.101 T > C, c.341A > G and c.608 T > C were polymorphisms. The category of two nucleotide changes (c.368C > A, c.571 T > C) was unknown (http://davinci.crg.es/deafness/).

**Table 1:** **Variations in *GJB2 *gene identified in all subjects**

**Nucleotide change**	**Amino acid change**	**Domain**	**Category**	**PolyPhen-2 prediction**	**SIFT prediction**	**Previous report**	**Numbers found in patients (N = 658)**	**Freq in patients (%)**	**Numbers found in controls (N = 462)**	**Freq in controls (%)**
c.35delG	Frameshift	IC1	Pathogenic	N/A	N/A	Yes	2	0.30	0	0.00
c.79G > A	Val27Ile	TM1	Polymorphism	1.000	0.15	Yes	135	20.52	65	14.07
c.101 T > C	Met34Thr	TM1	Polymorphism	0.038	0.12	Yes	3	0.46	1	0.22
c.109G > A	Val37Ile	TM1	Pathogenic	1.000	0.12	Yes	15	2.28	4	0.87
c.176del16	Frameshift	EC1	Pathogenic	N/A	N/A	Yes	21	3.19	2	0.43
c.235delC	Frameshift	TM2	Pathogenic	N/A	N/A	Yes	102	15.50	3	0.65
**c.257C > G**	**Thr86Arg**	**TM2**	**Pathogenic**	**1.000**	**0.00**	**None**	**2**	0.30	**0**	0.00
c.299delAT	Frameshift	IC2	Pathogenic	N/A	N/A	Yes	31	4.71	2	0.43
c.341A > G	Glu114Gly	IC2	Polymorphism	0.001	0.27	Yes	25	3.80	9	1.95
c.368C > A	Thr123Asn	IC2	Unknown	0.000	0.53	Yes	5	0.76	1	0.22
c.504insAAGG	Frameshift	EC2	Pathogenic	N/A	N/A	Yes	2	0.30	0	0.00
c.571 T > C	Phe191Leu	EC2	Unknown	1.000	0.00	Yes	4	0.61	0	0.00
c.605ins46	Stop at aa 202	TM4	Pathogenic	N/A	N/A	Yes	2	0.30	0	0.00
c.608TC > AA	Ile203Lys	TM4	Pathogenic	N/A	0.00	Yes	2	0.30	0	0.00
c.608 T > C	Ile203Thr	TM4	Polymorphism	0.906	0.00	Yes	2	0.30	1	0.22
**Total**							353	53.65	88	19.05

*IC* intracellular, *TM* transmembrane, *EC* extracellular.

The amino acid substitution is predicted damaging by the score of PolyPhen-2 (0 being least and 1 being most) and SIFT (ranges from 0 to 1, damaging < = 0.05, tolerated > 0.05).

The carrier frequencies of deafness-causing *GJB2* mutations in our case group were 0.30% for c.35delG, 2.28% for c.109G > A, 3.19% for c.176del16, 15.50% for c.235delC, 4.71% for c.299delAT, 0.30% for c.504insGCAA, 0.30% for c.605ins46 and 0.30% for c.608TC > AA, respectively. Thus, the total carrier frequency of pathogenic *GJB2* mutations in the case group was 24.92% (164/658). The 235delC appeared to be the most common deafness-causing *GJB2* mutation (102/658, 15.50% ) with the highest allele frequency. While in the control group, only eleven subjects were detected to carry the heterozygous deafness-causing mutations.

The detailed genotypes of the patients and controls were summarized as Table 2. Of all patients, 70 had monoallelic *GJB2* coding region mutations in the heterozygous state, 94 carried two confirmed pathogenic mutations including 79 homozygotes and 15 compound heterozygotes. The genotypes-phenotypes correlations of *GJB2* gene mutations in 658 patients were shown in Table 3. In the control group, we detected three c.109G > A, two c.176del16, three c.235delC and two c.299delAT heterozygotes, representing 2.38% of all normal hearing individuals, which coincided with previous study in different control cohorts. No mutations or variants in the *GJB2* exon1 and basal promoter region were found in both the case and control groups.

**Table 2:** **Genotypes of *GJB2 *gene in patients with hearing loss and controls**

**Allele 1**			**Allele 2**			**Numbers found in patients (N = 658)**	**Numbers found in controls (N = 462)**	**Exon 1 or splice site nucleotide change**
**Nucleotide change**	**Consequence or amino acid change**	**Category**	**Nucleotide change**	**Consequence or amino acid change**	**Category**			
c.35delG	Frameshift mutation	pathogenic	-	-	-	1	0	-
c.109G > A	Val37Ile	pathogenic	-	-	-	7	3	-
c.176del16	Frameshift mutation	pathogenic	-	-	-	6	2	-
c.235delC	Frameshift mutation	pathogenic	-	-	-	40	3	-
c.299delAT	Frameshift mutation	pathogenic	-	-	-	12	2	-
c.504insAAGG	Frameshift	pathogenic	-	-	-	2	0	-
c.608TC > AA	Ile203Lys	pathogenic	-	-	-	2	0	-
c.35delG	Frameshift mutation	pathogenic	c.235delC	Frameshift mutation	pathogenic	1	0	-
c.109G > A	Val37Ile	pathogenic	c.109G > A	Val37Ile	pathogenic	8	0	-
c.176del16	Frameshift mutation	pathogenic	c.176del16	Frameshift mutation	pathogenic	7	0	-
c.176del16	Frameshift mutation	pathogenic	c.235delC	Frameshift mutation	pathogenic	5	0	-
c.176del16	Frameshift mutation	pathogenic	c.299delAT	Frameshift mutation	pathogenic	3	0	-
c.235delC	Frameshift mutation	pathogenic	c.235delC	Frameshift mutation	pathogenic	52	0	-
c.235delC	Frameshift mutation	pathogenic	c.299delAT	Frameshift mutation	pathogenic	4	0	-
**c.257C > G**	**Thr86Arg**	**pathogenic**	**c.605ins46**	**Stop at aa 202**	**pathogenic**	**2**	**0**	-
c.299delAT	Frameshift mutation	pathogenic	c.299delAT	Frameshift mutation	pathogenic	12	0	-
c.79G > A	Val27Ile	polymorphism	-	-	-	64	28	-
c.101 T > C	Met34Thr	polymorphism	-	-	-	3	1	-
c.79G > A	Val27Ile	polymorphism	c.79G > A	Val27Ile	polymorphism	49	13	-
c.79G > A	Val27Ile	polymorphism	c.341A > G	Glu114Gly	polymorphism	9	1	-
c.341A > G	Glu114Gly	polymorphism	c.341A > G	Glu114Gly	polymorphism	5	1	
c.341A > G	Glu114Gly	polymorphism	c.79G > A					
c.341A > G	Val27Ile							
Glu114Gly	polymorphism							
polymorphism	2	0	-					
c.79G > A	Val27Ile	polymorphism	c.368C > A	Thr123Asn	unknown	11	3	-
c.608 T > C	Ile203Thr	polymorphism	c.608 T > C	Ile203Thr	polymorphism	4	0	-
c.368C > A	Thr123Asn	unknown	-	-	-	1	1	-
c.571 T > C	Phe191Leu	unknown	-	-	-	4	0	-

**Table 3:** ***GJB2 *genotypes and phenotypes in the 658 unrelated patients**

**Genotype**	**Number of subjects with this genotype**	**Phenotype**			**Onset**
		**moderate**	**severe**	**profound**	
*Biallelic mutations*	94				
c.35delG/c.235delC	1			1	Prelingual
c.109G > A/c.109G > A	8	2	2	4	Post (2), pre (6)
c.176del16/c.176del16	7		3	4	Prelingual
c.176del16/c.235delC	5		1	4	Prelingual
c.176del16/c.299delAT	3			3	Prelingual
c.235delC/c.235delC	52	1	11	40	Prelingual
c.235delC/c.299delAT	4			4	Prelingual
c.257C > G/c.605ins46	2			2	Prelingual
c.299delAT/c.299delAT	12	1	1	10	Prelingual
*Heterozygous mutations*	70				
c.35delG	1			1	Prelingual
c.109G > A	7	2	3	2	Post (3), pre (4)
c.176del16	6		1	5	Prelingual
c.235delC	40	3	8	29	Post (8), pre (32)
c.299delAT	12	1	3	8	Prelingual
c.504insAAGG	2		1	1	Prelingual
c.608TC > AA	2	1		1	Prelingual
*Polymorphism and unknown*	152				
c.79G > A	64	13	22	29	Post (3), pre (61)
c.101 T > C	3	1	1	1	Post (1), pre (2)
c.79G > A/c.79G > A	49	11	15	23	Post (5), pre (44)
c.79G > A/c.341A > G	9	1	1	7	Prelingual
c.341A > G/c.341A > G	5	2	2	1	Prelingual
c.341A > G/c.341A > G/c.79G > A	2		1	1	Prelingual
c.79G > A/c.368C > A	11	1	1	9	Prelingual
c.368C > A	1		1		Prelingual
c.571 T > C	4		2	2	Prelingual
c.608 T > C/c.608 T > C	4		1	3	Prelingual
*No mutations identified*	342	28	52	262	
**Total**	**658**				

### Mitochondrial 12S rRNA gene mutations

The two DNA fragments spanning the entire coding region of the *12S rRNA* gene were amplified by PCR from genomic DNA of all subjects, and each fragment was purified and subsequently analyzed by direct DNA sequencing. The sequencing results were compared with the updated Cambridge consensus sequence. As shown in Table 4, there were totally 30 kinds of nucleotide changes identified in the *12S rRNA* gene. All the nucleotide changes were verified by sequence analysis of both strands and appeared to be homoplasmy. Among these, four subjects with severe or profound hearing loss carried the m.1494C > T mutation. Three of them had a history of exposure to aminoglycosides before deafness. Thirty-nine patients harbored the m.1555A > G mutation. These translate to a frequency of 5.93% and 0.61% for the m.1555A > G and m.1494C > T mutations in deafness population of Jiangsu Province, respectively. Meanwhile, 6 subjects harbored the known deafness-associated m.1095 T > C mutation and 18 subjects carried the putative pathogenic mutations at position of 961 (m.961insC, m. 961delT and m.961 T > C).

**Table 4:** **Mitochondrial *12S rRNA *variants identified in this study**

**Nucleotide change**	**Homo/heteroplasmy**	**NSHL (N = 658)**	**Freq in patients(%)**	**Controls (N = 462)**	**Freq in controls(%)**	**conservation index**^**a**^	**Chinese (N = 1642)**^**b**^	**freq in Chinese(%)**	**Previous report**^**c**^	**mtDB**^**c **^**(N = 2704)**	**Freq in mtDB (%)**
663 A > G	homoplasmy	4	0.61	3	0.65	17/22(77.3% )	16	1.0	Yes	86	3.2
681 T > C	homoplasmy	9	1.37	6	1.30	18/22(81.8% )	30	1.8	Yes	11	0.4
709 G > A	homoplasmy	143	21.73	109	23.59	14/22(63.6% )	330	20.1	Yes	444	16.4
735 A > G	homoplasmy	4	0.61	2	0.43	16/22(72.7% )	10	0.6	Yes	3	0.1
750 A > G	homoplasmy	656	99.70	460	99.57	22/22(100% )	1638	99.8	Yes	2682	96.7
752 C > T	homoplasmy	16	2.43	15	3.25	22/22(100% )	51	3.1	Yes	20	0.7
789 T > C	homoplasmy	1	0.15	0	0.00	20/22(90.9% )	2	0.1	Yes	1	0.0
827 A > G	homoplasmy	24	3.65	13	2.81	20/22(90.9% )	54	3.3	Yes	54	2.0
961 insC	homoplasmy	15	2.28	1	0.22	20/22(90.9% )	25	1.5	Yes	37	1.4
961 delT + insC	both	2	0.30	0	0.00	20/22(90.9% )	1	0.1	Yes	no data	no data
961 T > C	homoplasmy	1	0.15	1	0.22	20/22(90.9% )	3	0.2	Yes	37	1.4
979C > T	homoplasmy	1	0.15	0	0.00	6/22(27.3% )	0	0.0	Yes	1	0.0
1005 T > C	both	29	4.41	22	4.76	9/22(40.9% )	72	4.4	Yes	7	0.3
1009 C > T	homoplasmy	4	0.61	3	0.65	3/22(13.6% )	10	0.6	Yes	2	0.1
1040 T > C	homoplasmy	1	0.15	0	0.00	8/22(36.4% )	0	0.0	Yes	2	0.1
1041 A > G	homoplasmy	6	0.91	1	0.22	7/22(31.8% )	11	0.7	Yes	14	0.5
1048 C > T	homoplasmy	17	2.58	11	2.38	13/22(59.1% )	48	2.8	Yes	51	1.9
1095 T > C	homoplasmy	6	0.91	1	0.22	22/22(100% )	10	0.6	Yes	5	0.2
1107 T > C	homoplasmy	46	6.99	29	6.28	18/22(81.8% )	103	6.3	Yes	34	1.3
1119 T > C	homoplasmy	23	3.50	19	4.11	13/22(59.1% )	53	3.2	Yes	26	1.0
1187 T > C	homoplasmy	3	0.46	1	0.22	11/22(50% )	0	0.0	Yes	1	0.0
1222A > G	homoplasmy	**1**	0.15	**0**	0.00	22/22(100% )	0	0.0	None	0	0.0
1282G > A	homoplasmy	2	0.30	0	0.00	13/22(59.1% )	0	0.0	Yes	2	0.1
1382A > C	homoplasmy	17	2.58	11	2.38	17/22(77.3% )	43	2.6	Yes	65	2.4
1415G > A	homoplasmy	1	0.15	0	0.00	8/22(36.4% )	1	0.1	Yes	1	0.0
1438A > G	homoplasmy	658	100.00	461	99.78	22/22(100% )	1640	99.9	Yes	2620	96.9
1494C > T	homoplasmy	4	0.61	0	0.00	18/22(81.8% )	3	0.2	Yes	1	0.0
1520 T > C	homoplasmy	3	0.46	0	0.00	6/22(27.3% )	6	0.4	Yes	3	0.1
1555A > G	homoplasmy	39	5.93	0	0.00	20/22(90.9% )	65	4.0	Yes	12	0.4
1598G > A	homoplasmy	14	2.13	10	2.16	22/22(100% )	49	3.0	Yes	67	2.5

^a^The conservation index (CI) was based on the results of the multiple alignment by ClustalW. See Additional file 1: Table S1 for information on the species used to calculate the sequence conservation; ^b^Data from the refrence [15]; ^c^Uppsala mtDB database [16].

**Figure 1:**
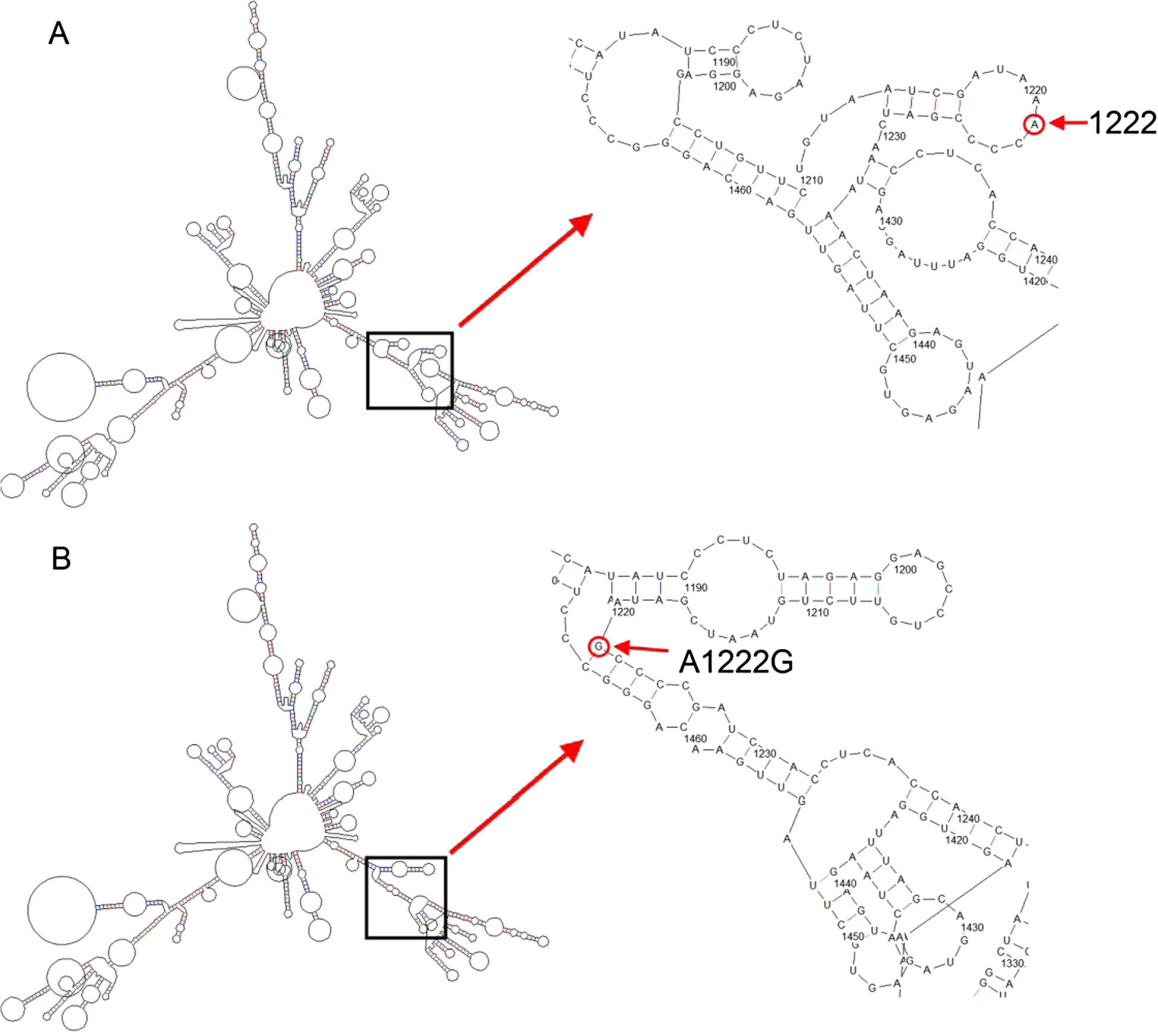
**12S rRNA secondary structures predicted by RNAdraw. (A)** wild-type 12S rRNA; **(B)** 12S rRNA with the m.1222A>G variant. To the right, which come from the corresponding panels, is shown an enlargement of the region of predicted secondary structures surrounding nucleotide positions 1222 (bold arrows with red circles).

A novel sequence variant, m.1222A > G in the *12S rRNA* gene, was identified in one patient with profound hearing loss. This variant was not found in 462 controls. Furthermore, we used RNAdraw software to analyze the secondary structure of 12S rRNA to localize m.1222A > G variant with either a stem or a loop and to analyze if the base changes within stems alter. As shown in Figure 1, the secondary structure of the 12S rRNA m.1222A > G variant predicted by RNAdraw indicated that the m.1222A > G induced a marked structural alteration in the transcript.

In addition, phylogenetic analysis was performed by comparing the human *12S rRNA* nucleotide variants with other 21 primates. As shown in Table 4, conservation index (CI) among the variants ranged from 13.6% (m.1009C > T variant) to 100% (m.750A > G, m.752C > T, m.1095 T > C, m.1222A > G, m.1438A > G and m.1598G > A variants). Of 30 variants, 15 had a CI more than 78% , 8 had a CI between 78% and 50% , and the remaining had CIs below 50% . In addition to the m.1555A > G and m.1494C > T mutations, the m.789 T > C and m.1222A > G variants, which were absent in the 462 controls and whose CIs were above 78% , were the putative deafness-associated variants. In contrast, other 9 variants including m.663A > G, m.681 T > C, m.750A > G, m.752C > T, m.827A > G, m.1107 T > C, m.1438A > G and m.1598G > A, which were present in both the case and control groups, appeared to be the polymorphisms even though they had CIs above 78%.

## Discussion

Hearing loss is the most common neurosensory disorder in humans. Approximately half of the cases have a genetic etiology with extraordinary genetic heterogeneity. For many populations, the most common cause for NSHL is mutated Connexin 26, a gap junction protein encoded by the *GJB2* gene, which is expressed in the cochlea and may play a role in K^+^ circulation between different partitions in the cochlea. *GJB2* gene is composed of 2 exons separated by an intron. Its coding region is entirely contained in exon 2. The basal promoter activity resides in the first 128 nucleotides upstream of the transcription start point (TSP) and has two GC boxes, at position 281 and 293 from the TSP, which is important for transcription [17]. Previous reports have suggested that the mutation spectrum and prevalence of *GJB2* vary significantly among different ethnic groups. *GJB2* mutations with the c.35delG, c.167delT, c.235delC and c.427C > T alleles were responsible for a large proportion of NSHL in European, American, African and the Asian [18-25]. In the Chinese populations, the 235delC is responsible for up to 21% of cases with NSHL [9].

Similar to other previous reports of China [10,14,26], our study showed that *GJB2* mutations were an important cause of NSHL in Jiangsu deafness populations, and the most common *GJB2* mutation detected in our study group was the c.235delC (102/658; 15.50% ). While the c.35delG mutation, which is prevalent in the Caucasians and rarely found in Chinese populations, was identified only in 2 patients. The four most prevalent mutations: c.235delC, c.299delAT, c.176del16, and c.35delG, account for 23.70% (156/658) of all mutant *GJB2* alleles identified in Jiangsu Province. However, no mutations were found in *GJB2* basal promoter region and exon 1 or splice sites, which suggested extremely low detection rate of *GJB2* basal promoter region and exon 1 or splice sites mutation in this area.

Through genotype analysis in 658 cases of unrelated NSHL of Jiangsu Province, *GJB2* mutations were detected in 14.29% (94/658) of patients with biallelic mutation, and 10.64% (70/658) of patients with monoallelic mutation. The reason for the failure to detect a second mutant allele in the 70 cases in the present study claims further clarification.

One novel nucleotide alteration c.257C > G (T86R) in coding region of *GJB2* gene were identified in two deaf siblings compound heterozygotes carrying c.605ins46 pathogenic mutation in the exon 2 of *GJB2*, this change has not been reported in Connexins and Deafness mutations database at http://davinci.crg.es/deafness/. The heterozygotes were detected from their hearing parents, the father carried c.257C > G (T86R) mutation, while the mother with c.605ins46 mutation, the opposite alleles of the parents were both wild-type.

Although the majority of cases with NSHL are caused by nuclear gene mutations, it has become clear that mtDNA mutations can also cause deafness. In familial cases of ototoxicity, aminoglycoside hypersensitivity is often maternally transmitted, suggesting that the mutation in mtDNA is the molecular basis for this susceptibility. In the human mtDNA genome, m.1555A > G is the most common mutation in the gene. The *12S rRNA* gene was proposed to be the primary targeting site for aminoglycosides. The identified nonsyndromic deafness-causing mtDNA mutations include m.1555A > G, m.1494C > T, m.1095 T > C and mutations at position 961 in the *12S rRNA* gene [27-32]. Currently, it is estimated that these mutations are present in about 3.10% of patients with NSHL, but it is expected that this number will increase as genetic testing becomes more readily available. In this study, 658 patients and all controls were screened for mtDNA *12S rRNA* gene by PCR and DNA direct sequencing. We detected 30 variants in *12S rRNA* gene. Among these, 39 patients carried the mtDNA *12S rRNA* m.1555A > G mutation (5.93% ), which coincided with the previous report [15]. Four patients carried m.1494C > T mutation, 6 patients carried m.1095 T > C mutation, and 18 patients possess mutations at 961 position. A novel sequence variant, m.1222A > G in the *12S rRNA* gene, was identified in one patient with profound hearing loss. Comparison of the variant frequencies in controls, assessment of nucleotide conservation among mammalian species, and structural analysis of the transcript were used to analyze the m.1222A > G mutation associated with hearing loss. To our knowledge, the homoplasmic m.1222A > G variant in *12S rRNA* gene has not been reported to date. Conservation of the nucleotide among 22 primates and alteration of the predicted secondary structure of the *12S rRNA* transcript suggest that the m.1222A > G variant might affect auditory function by changing the function of the 12S rRNA.

Similar to that of the *GJB2* gene mutations, the association between mtDNA mutations and hearing loss has also a special ethnic and regional difference. In the NSHL cases of Jiangsu population, the incidence of the mtDNA mutation including m.1555A > G, m.1494C > T, m.1095 T > C and mutations at position 961 in the *12S rRNA* gene appears to coincide with other populations as previous reports [15].

In summary, the results of our study indicate that the mutation spectrum and prevalence of *GJB2* and mtDNA *12S rRNA* genes in Jiangsu patients with hearing loss is similar to other areas of China. There are in total 31.46% of patients with NSHL carry deafness-causing mutations in *GJB2* or mtDNA *12S rRNA* gene. Mutation in *GJB2* gene was the most common factor, mtDNA *12S rRNA* also play an important part in the pathogenesis of hearing loss in Jiangsu Province areas. These results indicate the necessity of genetic screening for mutations of these genes in Jiangsu patients with NSHL.

## Competing interests

The authors declare that they have no competing interests.

## Authors’ contributions

XC, GQX and QJW conceived and designed the study. SW, JY, YJL and ZBC performed the experiments and analysis. QJW and SW wrote the original manuscript. XC and GQX contributed to revisions of the manuscript. All authors read and approved the final version.

## Supplementary Material

Additional file 1: Table S1 List of animal species and the accession numbers of the mtDNA (GenBank) used to calculate nucleotide conservation. Click here for file
