# Supplementary material for: Genetic mutations of GJB2 and mitochondrial 12S rRNA in nonsyndromic hearing loss in Jiangsu Province of China
Source: J Transl Med. 2013 Jul 4;11:163. doi: 10.1186/1479-5876-11-163 (PMC3706284; doi:10.1186/1479-5876-11-163)
Supplement: Additional file 1: Table S1 — List of animal species and the accession numbers of the mtDNA (GenBank) used to calculate nucleotide conservation. [file 1479-5876-11-163-S1.doc]

**Table S1**

| *Homo sapiens* | NC_012920 |
| --- | --- |
| *Gorilla gorilla* | NC_001645 |
| *Pan paniscus* | NC_ 001644 |
| *Pan troglodytes* | NC_001643 |
| *Pongo pygmaeus* | NC_001646 |
| *Pongo abelii* | NC_002083 |
| *Hylobates lar* | NC_002082 |
| *Macaca mulatta* | NC_005943 |
| *Macaca sylvanus* | NC_002764 |
| *Papio hamadryas* | NC_001992 |
| *Cebus albifrons* | NC_002763 |
| *Tarsius bancanus* | NC_002811 |
| *Lemur catta* | NC_004025 |
| *Daubentonia madagascariensis* | NC_010299 |
| *Nycticebus coucang* | NC_002765 |
| *Tarsius syrichta* | NC_012774 |
| *Loris tardigradus* | NC_012763 |
| *Perodicticus potto* | NC_012764 |
| *Aotus lemurinus* | NC_019799 |
| *Gorilla gorilla* | NC_011120 |
| *Macaca fascicularis* | NC_012670 |
| *Hylobates klossii* | HQ_622788 |
